# ﻿Two new species and a new record of Comesomatidae (Nematoda, Araeolaimida) from the Southern Ocean

**DOI:** 10.3897/zookeys.1244.135491

**Published:** 2025-07-08

**Authors:** Sujing Fu, Jianfeng Mou, Kun Liu, Shuyi Zhang, Heshan Lin

**Affiliations:** 1 Third Institute of Oceanography, Ministry of Natural Resources, Xiamen, 361005, China Third Institute of Oceanography, Ministry of Natural Resources Xiamen China

**Keywords:** Biodiversity, free-living marine nematodes, *Hopperiabeaglense*, molecular phylogeny, *Sabatieriacosmonautae* sp. nov., *Sabatieriacrassilonga* sp. nov., taxonomy

## Abstract

Two new marine nematode species of the genus *Sabatieria* and a new record of *Hopperiabeaglense* Chen & Vincx, 1998 belonging to the family Comesomatidae are described from the Southern Ocean using morphological and molecular data. *Sabatieriacosmonautae***sp. nov.** is characterized by body length 1786–2230 μm, short cephalic setae, lateral differentiation of body cuticle with sparse punctations starting from amphid to near tail tip, spiral amphidial fovea with three turns, spicules arcuate and 1.2–1.5 cloacal body diameters long, with straight dorso-caudal gubernacular apophyses, and 16 or 17 precloacal supplements. *Sabatieriacrassilonga***sp. nov.** is characterized by body length 2628–3613 μm, short cephalic setae, cuticle with lateral differentiation of coarser and irregularly spaced punctations extending from anterior edge of amphid to the tail region, amphidial fovea spiral with 2.5 turns, males with spicules 1.2–1.5 cloacal body diameters long, gubernaculum with a long straight dorso-caudal apophysis, and 20 or 21 fine precloacal supplements. *Hopperiabeaglense*, which was originally described from the Strait of Magellan and the Beagle Channel, is recorded from the Southern Ocean for the first time. Phylogenetic analysis based on concatenated 18S + 28S rRNA sequences places the two new species and the new record within Comesomatidae. The tree topology shows Comesomatidae forming a well-supported monophyletic clade, whereas the genus *Sabatieria* is not monophyletic.

## ﻿Introduction

The family Comesomatidae Filipjev, 1918 is found from the intertidal zone and shallow sea areas to the deep sea, showing high dominance in marine sediments (Jensen 1979). The genus *Sabatieria* de Rouville, 1903 is one of the most common genera in Antarctica (Baishnab et al. 2023). Free-living marine nematodes have been used as indicators of biological, environmental, or physical disturbance, and *Sabatieria* is among the most tolerant genera to environmentally damaging factors (Ridall and Ingels 2021). Eighty-two valid *Sabatieria* species have been described to date; they are divided into five species groups, including the *praedatrix* group (50 species), the *armata* group (7 species), the *pulchra* group (9 species), the *celtica* group (7 species), the *ornata* group (7 species) and two ungrouped species (Platt 1985; Yang et al. 2019; Fu et al. 2023; Leduc and Zhao 2023). *Praedatrix* species group: most species with lateral cuticle differentiation of larger, more widely spaced punctations and amphids with three turns, but two or four turns may also occur. Spicules without central lamella distinct from the internal projection from proximal end. Simple tubular or pore-like supplements and straight gubernacular apophyses. This large group is relatively loosely defined, and unlike the other groups it is not characterized by any autapomorphic features. *Armata* species group: similar to *praedatrix* group except for the elongated cephalic setae (> 1.7 corresponding body diameter) and mostly slender bodies (a > 65). Amphids usually with three turns but four turns may also occur. Simple tubular supplements. *Pulchra* species group: pairs of short and relatively stout cervical setae present. Amphids with three to four turns. Five to nine conspicuous precloacal supplements. Gubernaculum with median pieces. *Celtica* species group: amphids with three turns. Body relatively large and stout. All have curved gubernacular apophyses, and conspicuous precloacal supplements. *Ornata* species group: amphids usually with three turns. Similar to *celtica* group except for the presence of a posterior group of more closely situated precloacal supplements (Platt 1985).

Studies on nematodes in the Southern Ocean began at the end of the 19^th^ century (von Linstow 1891). To date, more than 160 species of marine nematodes have been described; however, data on marine nematode taxonomic information in the Southern Ocean is still fragmentary (Allgén 1959; Platt 1983; Ingels et al. 2014; Leduc 2014; Leduc 2016; Shimada et al. 2017, 2019, 2021; Fu et al. 2023). Technological advances, including scanning microscopy and analysis of molecular data, have enhanced our understanding of biodiversity and the phylogenetic position of marine nematodes (Fonseca et al. 2017). In the present study, we describe two new *Sabatieria* species and a new record of *Hopperiabeaglense* Chen & Vincx, 1998 from the Southern Ocean using morphological and molecular data of 18S rRNA and D2-D3 expansion fragments of 28S rRNA. The new species and new record were found during a marine nematode diversity study resulting from the 37^th^ and 38^th^ Chinese Antarctic Research Expedition.

## ﻿Material and methods

### ﻿Sampling and sample processing

**Figure 1.**
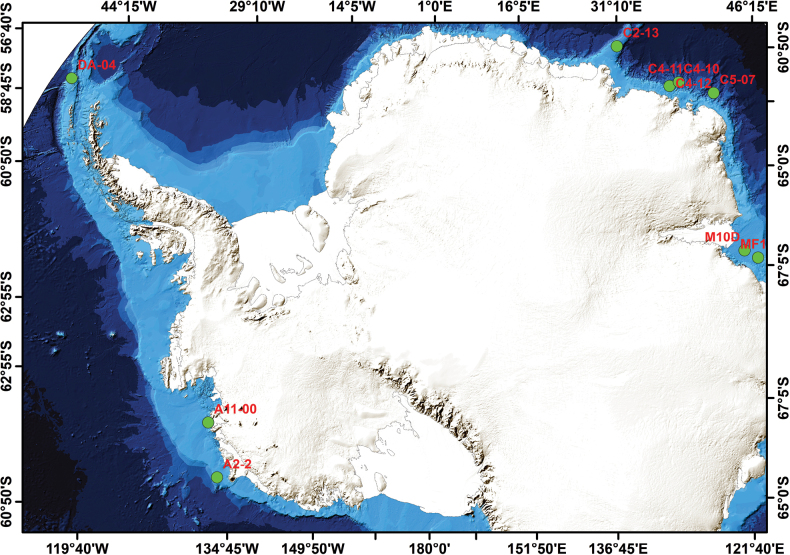
Map of sampling stations in the Southern Ocean. Green circles indicate sampling stations and red text indicates station names.

Sampling was conducted in the Antarctic Peninsula, the Cosmonauts Sea, Prydz Bay and Amundsen Sea during the 37^th^ and 38^th^ Chinese National Antarctic Research Expedition in the summer of 2021 and 2022 (Fig. 1, Table 1). As mentioned in our previous paper (Fu et al. 2023), the nematodes were sampled in the field, sorted, and slide-mounted in the laboratory.

**Table 1.** Sampling station details in the Southern Ocean.

Station	Latitude, Longitude	Region	Date	Depth (m)
DA-04	61.0046°S, 54.6971°W	Antarctic Peninsula	28 Dec. 2022	574
C4-12	67.1217°S, 44.3743°E	Cosmonauts Sea	22 Jan. 2021	1321
C4-11	66.5038°S, 45.0088°E	Cosmonauts Sea	18 Jan. 2021	2085
C4-10	66.5040°S, 45.0090°E	Cosmonauts Sea	20 Jan. 2021	2444
C5-07	65.3262°S, 49.9987°E	Cosmonauts Sea	20 Feb. 2022	2096
C2-13	67.2478°S, 33.2992°E	Cosmonauts Sea	26 Jan. 2021	1186
M10D	68.2833°S, 75.5827°E	Prydz Bay	31 Dec. 2020	578
MF1	67.5388°S, 77.3473°E	Prydz Bay	1 Jan. 2021	310
A2-2	72.7167°S, 124.5031°W	Amundsen Sea	7 Feb. 2022	457
A11-00	73.9433°S, 112.3525°W	Amundsen Sea	5 Feb. 2022	773

The observations were conducted using differential interference contrast microscopy (LEICA DM6B), and line drawings were made with a camera lucida. For scanning electron microscopy (SEM), specimens were placed in a 2% osmium tetroxide solution overnight, then dehydrated through a graded ethanol series, and critical point drying. Finally, individuals were mounted onto stubs before coating with gold using a sputter coater. Observations were made using a focused ion beam SEM (Helios 5 UC) at Xiamen University.

All measurements are given in μm and curved structures measured along the arc. All holotypes and paratypes are deposited in the Marine Biological Sample Museum of the Chinese Offshore Investigation and Assessment (**MBSMCOIA**) at the Third Institute of Oceanography, Ministry of Natural Resources, China.

Abbreviations are as follows: **a**, body length/maximum body diameter; **b**, body length/pharynx length; **c**, body length/tail length; **c**’, tail length/cloacal body diameter; **cbd**, corresponding body diameter; **L**, total body length; **n**, number of specimens; **V**, vulva distance from anterior end of body; **V**%, V/total body length × 100.

### ﻿DNA extraction, PCR amplification, and sequencing

Sediment samples used for molecular analysis were stored at -20 °C. Specimens of the two new species and one record were mounted on separate temporary slides to be photographed for morphological identification. The DNA extraction of each specimen was done using the Animal Genomic DNA Quick Extraction Kit for PCR analysis produced by Beyotime (Shanghai, China). Two fragments of 18S rRNA and 28S rRNA genes were amplified. For 18S rRNA of *Sabatieriacosmonautae* sp. nov. and *Sabatieriacrassilonga* sp. nov., 18SFi (5’-TGAATAACTACGCCGATCGCA-3’), and the reverse primer 18Sri (5’-CGAGCTTATGACCCACACTTACTG-3’) were designed using Primer Premier 5. For 28S rRNA, D2A (5′-ACAAGTACCGTGAGGGAAAGT-3′) and the reverse primer D3B (5′-TGCGAAGGAACCAGCTACTA-3′) were used following Nunn (1992). For both 18S rRNA and 28S rRNA, we used the 25-μL polymerase chain reaction (PCR) based on the method of our previous paper (Fu et al. 2023). Sequencing reactions were performed by Sangon Biotech (Shanghai) Co., Ltd. (Shanghai, China).

### ﻿Sequence alignment and phylogenetic analyses

The DNA sequences of *Sabatieriacosmonautae* sp. nov. (18S rRNA and 28S rRNA), *Sabatieriacrassilonga* sp. nov. (18S rRNA and 28S rRNA) and *Hopperiabeaglense* were deposited in GenBank and the accession numbers are PP693307 and PP693308, PP693305 and PP693306, PP693309 and PP693358, respectively. Sequences used in the phylogenetic analysis were based on a single specimen of three species from three genera in the family Axonolaimidae, Filipjev, 1918 (outgroup) and 13 species from five genera in the family Comesomatidae, which were longer than 900 bp for 18S rRNA and 600 bp for 28S rRNA.

PhyloSuite (Zhang et al. 2020) was utilized to conduct the analyses using the following programs: sequences were aligned using MAFFT (Katoh et al. 2002) with default parameters; TrimAl v. 1.2 (Capella-Gutiérrez et al. 2009) was used to remove ambiguously aligned sequences with default settings; and ModelFinder (Kalyaanamoorthy et al. 2017) was used to select the best-fit model based on the corrected Akaike information criterion (AICc) (Sugiura 1978).

Phylogenetic analyses were carried out using Bayesian inference (BI) methods and maximum likelihood (ML) methods. Bayesian inference phylogenies were inferred using MrBayes v.3.2.6 (Ronquist et al. 2012). The best-fit model for the two new species and one new record for 18S rRNA was found to be GTR+F+I+G4, with two parallel runs for 2000000 generations; for 28S rRNA, it was under GTR+F+G4 with two parallel runs for 2000000 generations. Maximum likelihood phylogenies were performed using IQ-TREE (Nguyen et al. 2015). The best-fit model for the two new species and one new record for 18S rRNA was found to be under TIM2e+I+I+R2 for 10000 bootstraps; for 28S rRNA, it was under TIM3e+R2 for 10000 bootstraps. The results were viewed and embellished in iTOL v.6 (Letunic and Bork 2016). Genetic distances (p-distance) over sequence pairs were calculated in MEGA X (Kumar et al. 2018).

## ﻿Results

### ﻿Systematics

**Family Comesomatidae Filipjev, 1918**

**Subfamily Sabatieriinae Filipjev, 1934**

#### ﻿Genus Sabatieria

Taxon classificationAnimaliaAraeolaimidaComesomatidae

de Rouville, 1903

4F42972F-7F22-5AFE-8608-1D79294AEF1D

##### Diagnosis

**(modified from Fonseca and Bezerra (2014)).** Cuticle with transverse punctation, lateral differentiation of larger regular or irregular punctations may occur. Cephalic sensilla in three distinct circles, cephalic setae longer than the outer labial setae. Anterior buccal cavity cup-shaped, posterior portion narrow, weakly cuticularized. Amphid multi-spiral. Apophyses usually directed dorso-caudally or caudally. Precloacal supplements usually present.

#### ﻿ Sabatieria cosmonautae sp. nov.

Taxon classificationAnimaliaAraeolaimidaComesomatidae

07A15E44-0C79-530F-B4F9-CC26CB4A8587

https://zoobank.org/A4EFBA85-06CF-4C0E-9900-218F34095E86

**Figure 2.**
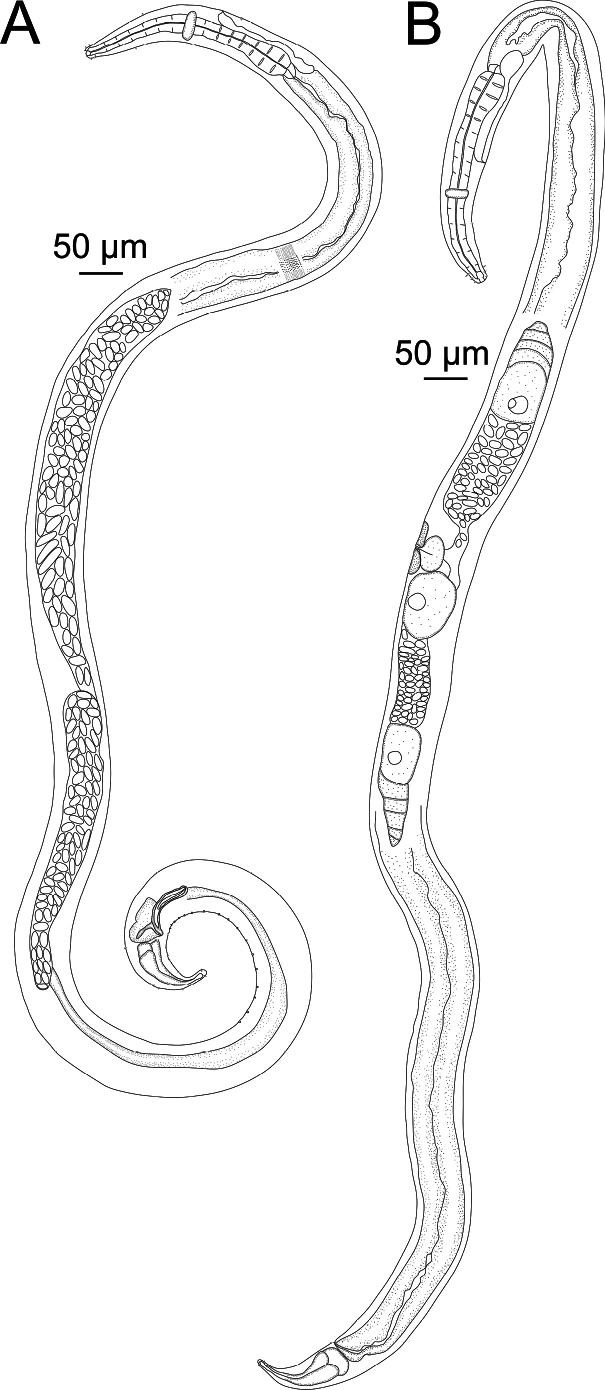
*Sabatieriacosmonautae* sp. nov. **A.** Entire male holotype; **B.** Entire female paratype.

**Figure 3.**
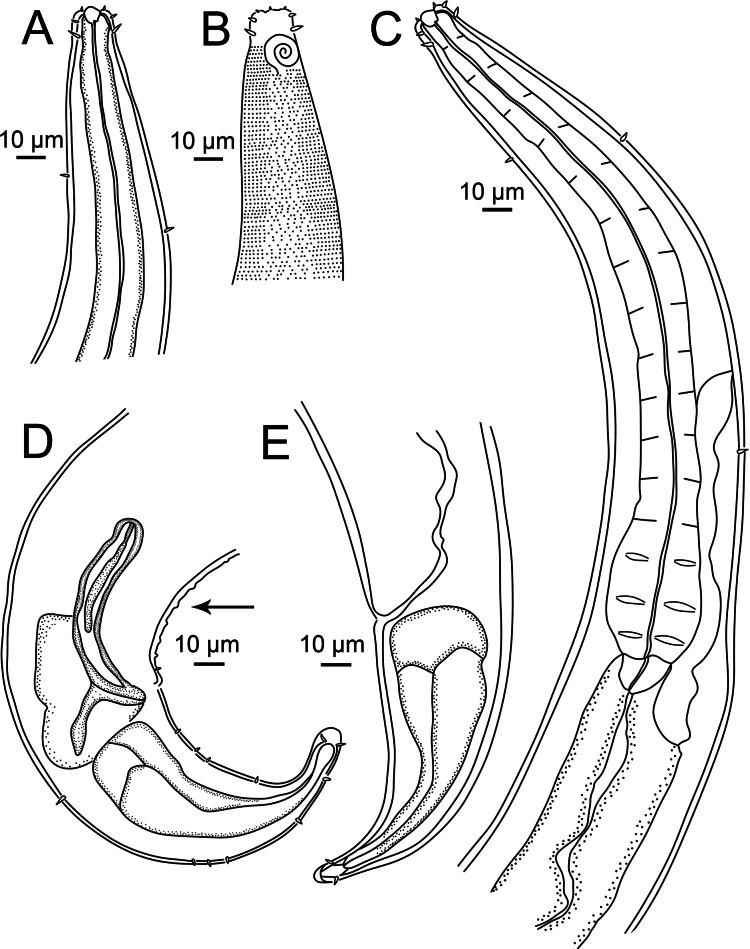
*Sabatieriacosmonautae* sp. nov. **A.** Male holotype anterior body region; **B.** Surface view of male holotype anterior body region showing amphidial fovea and cephalic sensilla; **C.** Male holotype pharyngeal body region; **D.** Male holotype posterior body region, showing precloacal supplements (arrow); **E.** Female paratype posterior body region.

**Figure 4.**
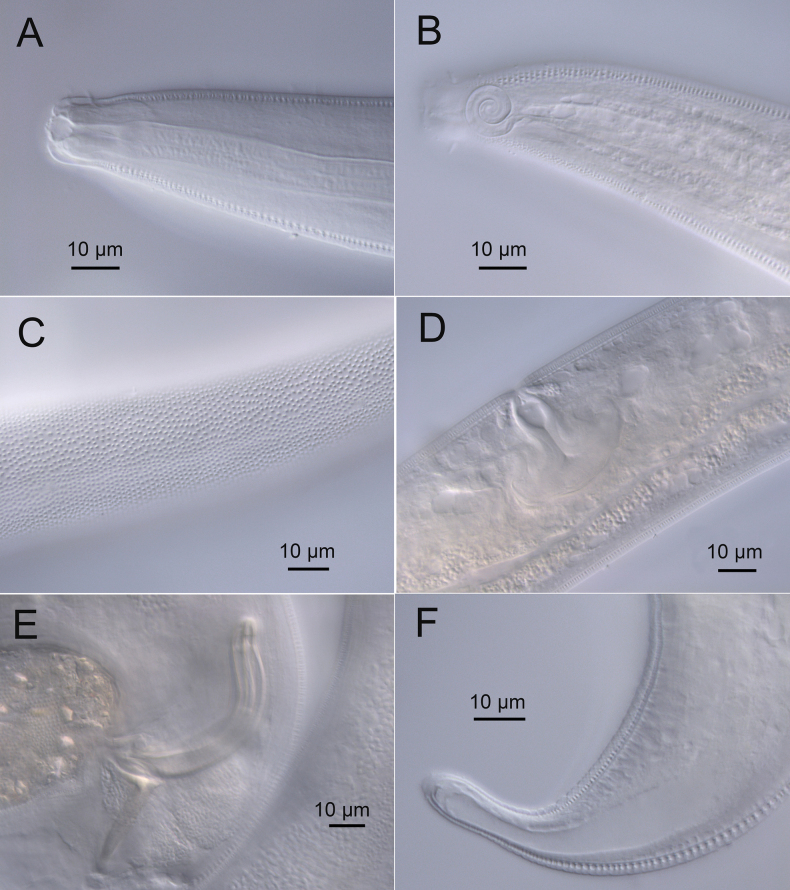
*Sabatieriacosmonautae* sp. nov. Light micrographs. **A.** Male paratype anterior body region showing the buccal cavity; **B.** Surface view of female paratype anterior body region showing amphids; **C.** Lateral punctations of male paratype anterior body region; **D.** Vulva; **E.** Male paratype posterior body region; **F.** Male paratype tail.

**Figure 5.**
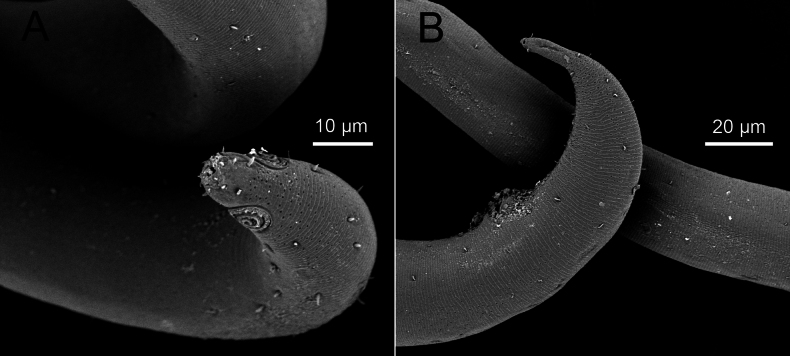
*Sabatieriacosmonautae* sp. nov. Scanning electron micrographs. **A.** Male anterior body region showing cephalic sensilla; **B.** Male posterior body region.

Figs 2 , 3 , 4 , 5 , Table 2

##### Material examined.

***Holotype***: • male, collected in the Cosmonauts Sea; 67.1217°S, 44.3743°E; depth 1321 m; collected on 22 Jan. 2021; Jianfeng Mou leg.; total organic carbon 0.22%; sand, 50.73%; clay, 7.62%; silt 41.11%; mean particle diameter 4.49 mm; on slide no. MBSMCOIA-C4-12-0-1-2. ***Paratypes***: • four males and three females, collected in the Cosmonauts Sea; 65.3262°S–67.1217°S, 44.3743°E–49.9987°E; depth 1321–2444 m; collected on 18 Jan. 2021–22 Jan. 2021, Feb. 2022; Jianfeng Mou leg.; total organic carbon 0.22%–0.61%; sand, 4.78%–50.73%; clay, 7.62%–23.70%; silt, 41.11%–71.51%; mean particle diameter 4.49–6.77 mm; males on slide no. MBSMCOIA-C5-7-0-2-2, MBSMCOIA-C4-10-0-2-2, MBSMCOIA-C4-11-2-4 and MBSMCOIA-C4-12-0-1; females on slide no. MBSMCOIA-C4-11-2-4.

##### Measurements.

All measurement data are given in Table 2.

**Table 2.** Morphometrics (μm) of *Sabatieriacosmonautae* sp. nov. from the Southern Ocean.

	Male holotype	Male paratypes	Female paratypes
Number of specimens	1	4	3
L	2230	1898 (1786–2009)	2123 (2032–2224)
a	35	29 (27–31)	35 (29–39)
b	9	8 (7–8)	8
c	18	16 (15–18)	18 (17–20)
c′	2.4	2.2 (1.9–2.4)	2.5 (2.4–2.6)
Head diameter at cephalic setae	14	16 (15–18)	15 (14–16)
Length of cephalic setae	4	3	3 (3–4)
Amphid height	13	12 (12–14)	11 (11–12)
Amphid width	12	12 (10–12)	11 (10–11)
Amphid width/cbd (%)	66	62 (54–68)	51 (48–54)
Amphid from anterior end	9	9 (8–10)	10 (9–12)
Nerve ring from anterior end	142	109 (94–125)	112 (102–128)
Nerve ring cbd	45	44 (41–47)	43 (41–45)
Excretory pore from anterior end	154	143 (126–162)	166 (149–184)
Pharynx length	254	250 (239–261)	264 (254–269)
Pharynx bulb cbd	54	55 (50–59)	53 (52–55)
Pharyngeal bulb diameter	30	34 (33–35)	38 (37–39)
Maximum body diameter	63	65 (59–73)	62 (57–69)
Spicule length	76	76 (71–79)	-
Gubernacular apophyses length	26	24 (21–29)	-
Number of precloacal supplements	17	16 or 17	-
Anal or cloacal body diameter	50	53 (50–58)	46 (46–47)
Tail length	122	116 (110–124)	116 (113–118)
V	-	-	1046 (1005–1082)
V%	-	-	49 (49–50)
Vulva body diameter	-	-	61 (57–69)

##### Description.

**Male.** Body short, narrowing gradually towards both extremities. Cuticle with transverse rows of punctations starting from amphid to near tail tip. Lateral differentiation with slightly sparse punctations. Cuticle striations distinct on cuticle surface using SEM (Fig. 5). Short somatic setae sparsely present on entire body, ~2 μm long. Cephalic region with distinct constriction at level of cephalic setae. Anterior sensilla in three crowns: six small inner labial papillae, six outer labial setae (2 μm long), and four cephalic setae (3 μm long or 0.15–0.25 corresponding body diameter long). Anterior buccal cavity cup-shaped, 5–6 μm wide, posterior portion narrow. Spiral amphidial fovea with three turns, 10–12 μm in diameter or 54–68% of corresponding body diameter, anterior border located at the level of cephalic setae. Pharynx surrounds half of anterior buccal cavity, gradually broadening posteriorly and forming a weak posterior bulb. Nerve ring situated at 39–56% of anterior pharynx. Secretory-excretory gland located posterior to pharynx. Secretory-excretory pore located posterior to nerve ring, 126–162 μm from the anterior end. Cardia small, completely surrounded by intestinal tissue. Reproductive system diorchic, anterior outstretched testis to the left of intestine and posterior outstretched testis to the right of the intestine. Spicules arcuate, equal, 1.2–1.5 cloacal body diameters long, proximal part of spicule with central cuticularized projection (lamella) extending from proximal end to approximately half of spicule. Gubernaculum with straight dorso-caudally directed apophyses. One precloacal seta 2 μm long. Sixteen or seventeen precloacal supplements, in the form of small pores, with distance between the anterior eight supplements (12–21 μm) irregularly distributed, posterior eight supplements with distance between adjacent supplements (5–13 μm) increasing towards anterior (Fig. 3D). Conico-cylindrical tail 1.9–2.4 cloacal body diameters long bearing three terminal setae, with very slightly swollen tip. Cylindrical part ~1/5 of total tail length. Three caudal glands present. Spinneret present.

**Female.** Similar to males. Reproductive system with two opposed, outstretched ovaries, with anterior ovary to the left of intestine and posterior ovary to the right of intestine. Spermatheca present. Vulva at 49–50% of body length from anterior. Granular vaginal glands present. Three caudal glands present.

##### Diagnosis and relationships.

*Sabatieriacosmonautae* sp. nov. is characterized by body length 1786–2230 μm, short cephalic setae, 0.15–0.25 corresponding body diameter long; cuticle with transverse rows of punctations starting from amphid to near tail tip; lateral differentiation with sparse punctations; spiral amphidial fovea with three turns; males with arcuate spicules, 1.2–1.5 cloacal body diameters long, straight dorso-caudally directed gubernacular apophyses, and with 16 or 17 precloacal supplements, in the form of small pores; Conico-cylindrical tail 1.9–2.4 cloacal body diameters long in males and 2.4–2.6 cloacal body diameters long in females.

*Sabatieriacosmonautae* sp. nov. belongs to the *praedatrix* group: amphids with three turns, the presence of straight gubernacular apophyses and with 16 or 17 pore-like precloacal supplements. There are 50 valid species in this group. Within the *praedatrix* group, S. *cosmonautae* sp. nov. most closely resembles *S.ancudiana* Wieser, 1954, *S.granifer* Wieser, 1954, *S.intermissa* Wieser, 1954, *S.lawsi* Platt, 1983, *S.parabyssalis* Wieser, 1954 as well as *S.praedatrix* de Man, 1907, which are all characterized by having nearly 17 precloacal supplements. In addition, the new species differs from *S.ancudiana* in the stouter body shape (a = 27–35 vs 42–65 in *S.ancudiana*), shorter cephalic setae (0.15–0.25 vs 0.60–0.70 cbd in *S.ancudiana*) and shorter tail (1.9–2.4 vs 3.5 cloacal body diameters in *S.ancudiana*), from *S.granifer* in the shorter tail (1.9–2.4 vs 3.3–4.8 cloacal body diameters in *S.granifer*), from *S.intermissa* in the stouter body shape (a = 27–35 vs 40–48 in *S.intermissa*), shorter cephalic setae (0.15–0.25 vs 0.70–0.90 cbd in *S.intermissa*), shorter spicules (1.2–1.5 vs 2.0 cloacal body diameters in *S.intermissa*) and shorter tail (1.9–2.4 vs 3.5 cloacal body diameters in *S.intermissa*), from *S.lawsi* in the shorter tail (1.9–2.4 vs 3.1–3.8 cloacal body diameters in *S.lawsi*), from *S.parabyssalis* in the shorter cephalic setae (0.15–0.25 vs 0.70 cbd in *S.parabyssalis*), and shorter tail (1.9–2.4 vs 3.8–4.3 cloacal body diameters in *S.parabyssalis*), and from *S.praedatrix* in the much shorter tail (1.9–2.4 vs 4.0–4.5 cloacal body diameters in *S.praedatrix*).

This new species is also similar to *S.bathycopia* Leduc, 2013 in the *celtica* group by the relative cephalic setae length, relative spicule length and the value of c'. The new species differs from *S.bathycopia* by the value of c' (1.9–2.4 vs 3.6–4.3 in *S.bathycopia*), the structure of spicules (without velum or swollen distal portion vs with velum and swollen distal portion in *S.bathycopia*), and the gubernaculum shape (straight vs curved in *S.bathycopia*).

##### Etymology.

This species is named after the type locality belonging to the Cosmonauts Sea.

#### ﻿ Sabatieria crassilonga sp. nov.

Taxon classificationAnimaliaAraeolaimidaComesomatidae

5C595496-BF9F-566D-B9E1-712B93EFCC7F

https://zoobank.org/5F2C0763-5B31-4041-91C7-CD08224DA181

**Figure 6.**
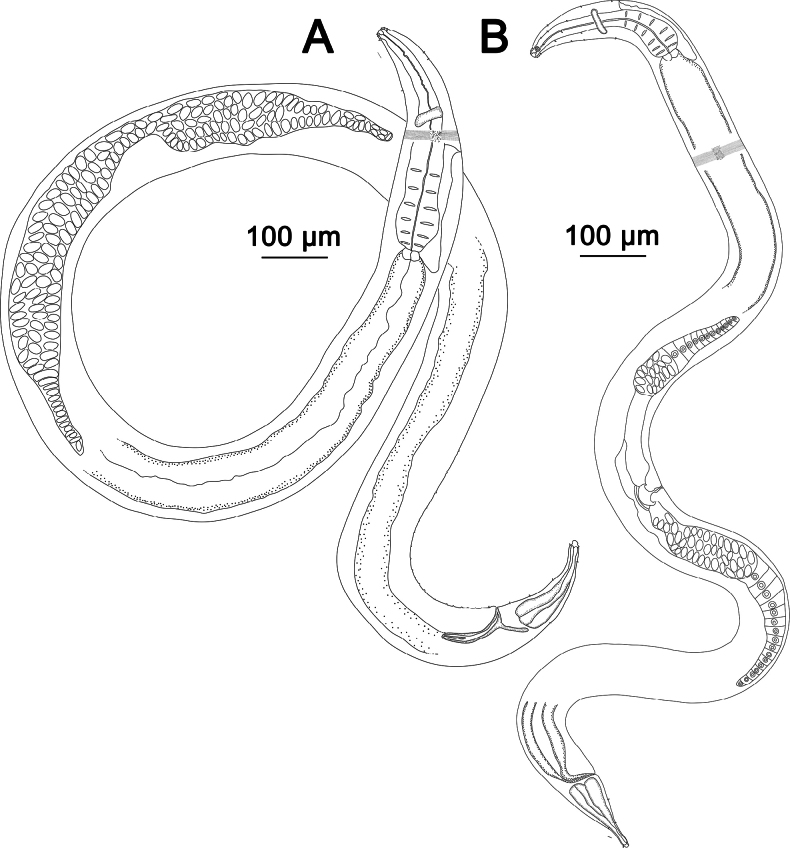
*Sabatieriacrassilonga* sp. nov. **A.** Entire male holotype; **B.** Entire female paratype.

**Figure 7.**
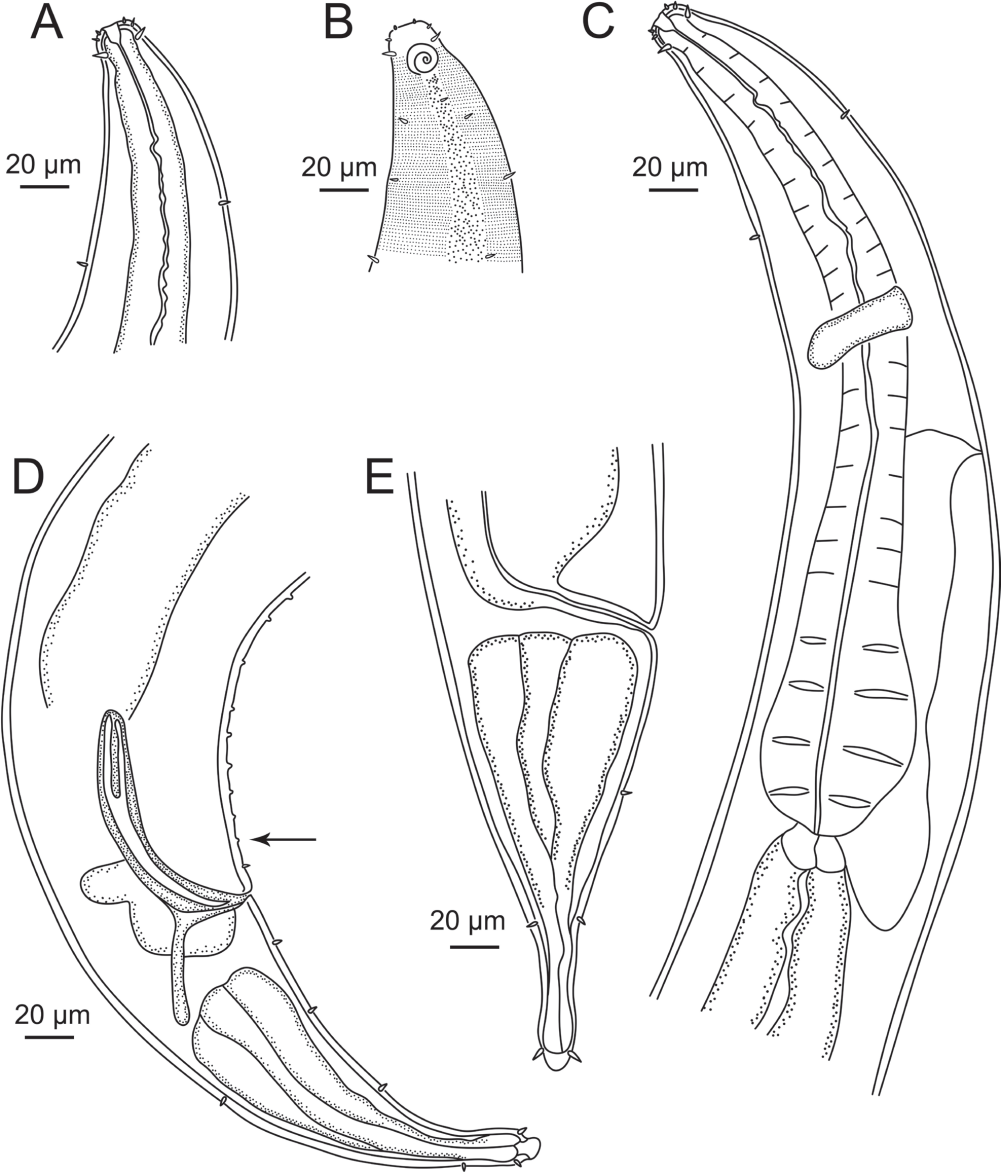
*Sabatieriacrassilonga* sp. nov. **A.** Male holotype anterior body region; **B.** Surface view of male holotype anterior body region showing amphidial fovea and cephalic sensilla; **C.** Male holotype pharyngeal body region; **D.** Male holotype posterior body region, showing precloacal supplements (arrow); **E.** Female paratype posterior body region.

**Figure 8.**
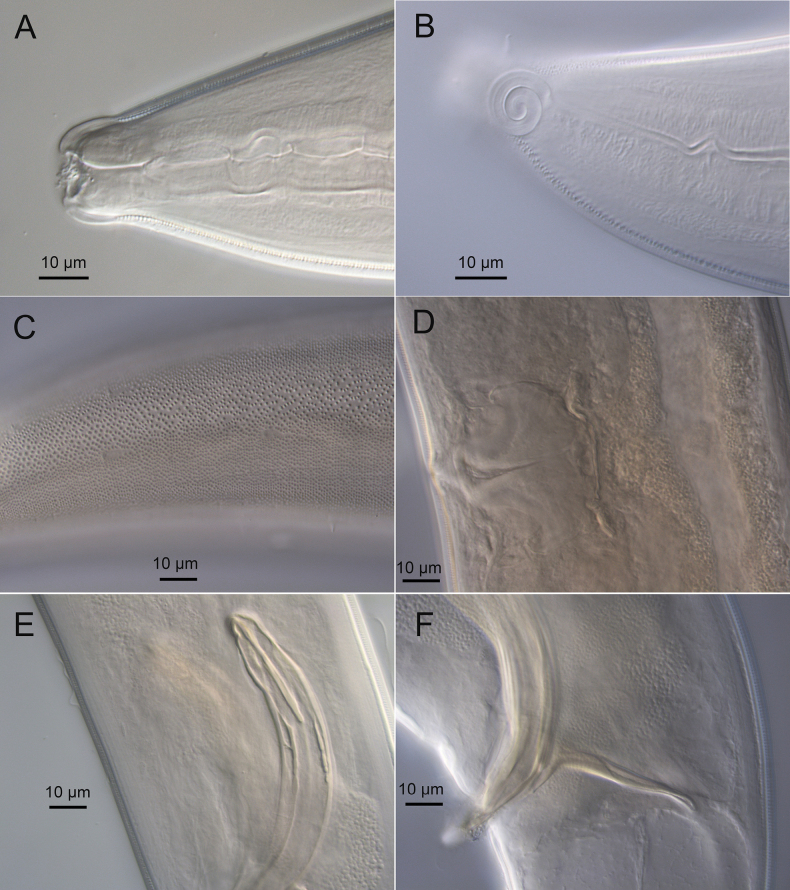
*Sabatieriacrassilonga* sp. nov. Light micrographs. **A.** Male paratype anterior part showing buccal cavity; **B.** Surface view of male paratype anterior body region showing amphids; **C.** Lateral punctations of female paratype anterior body region; **D.** Female paratype vulva; **E.** Male paratype spicule; **F.** Male paratype gubernaculum.

**Figure 9.**
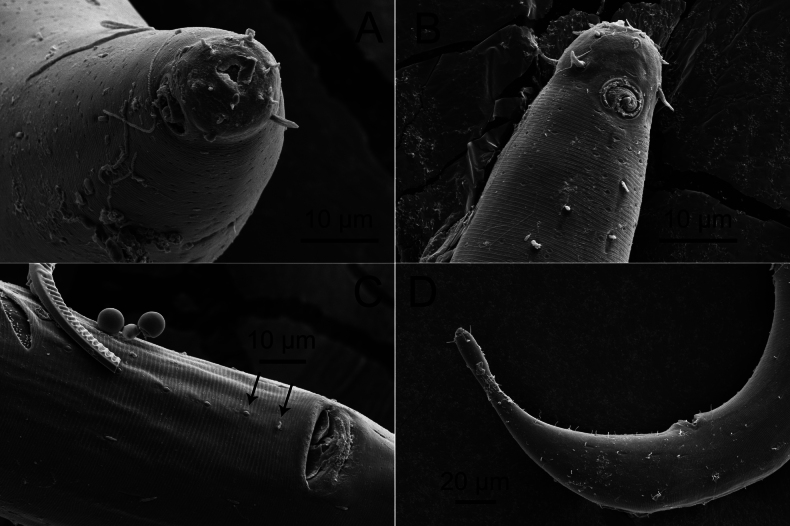
*Sabatieriacrassilonga* sp. nov. Scanning electron micrographs. **A.** Male anterior part showing cephalic sensilla and amphids; **B.** Male anterior part showing amphids; **C.** Male posterior part showing cloaca, precloacal seta (right arrow) and precloacal supplements (left arrow); **D.** Male posterior body region.

Figs 6 , 7 , 8 , 9 , Table 3

##### Material examined.

***Holotype***: • male, collected in the Amundsen Sea, 72.7167°S, 124.5031°W; depth 457 m; collected on 7 Feb. 2022; Jianfeng Mou leg.; muddy sediment; on slide no. MBSMCOIA-A2-2-2-5. ***Paratypes***: • three males and three females, collected in the Cosmonauts Sea, Prydz Bay and Amundsen Sea; 66.5038°S–73.9433°S, 33.2992°E–112.3525°W; depth 310-2085 m; collected on 1 Jan. 2021–22 Jan. 2021, Feb. 2022; Jianfeng Mou leg.; total organic carbon 0.12%–0.61%; sand, 4.78%–55.92%; clay, 7.62%–23.70%; silt, 35.58%–71.51%; mean particle diameter 4.36–6.77 mm; males on slide no. MBSMCOIA-C2-13-2-5-3, MBSMCOIA-A11-00-5-10-3, and MBSMCOIA-MF1-2-2; females on slide no. MBSMCOIA-MF1-2-2, MBSMCOIA-C4-11-2-4, and MBSMCOIA-C4-12-0-1.

##### Measurements.

All measurement data are given in Table 3.

**Table 3.** Morphometrics (μm) of *Sabatieriacrassilonga* sp. nov. from the Southern Ocean.

	Male holotype	Male paratypes	Female paratypes
Number of specimens	1	3	3
L	3093	2888 (2628–3085)	3176 (2910–3613)
a	30	28 (23–31)	27 (24–33)
b	9	8 (8)	8 (8)
c	16	17 (16–18)	17 (16–18)
c′	2.7	2 (1.8–2.1)	2.3 (2.1–2.4)
Head diameter at cephalic setae	20	21 (18–23)	24 (22–26)
Length of cephalic setae	4	5 (4–6)	5 (4–5)
Amphid height	12	12 (12–13)	12 (11–12)
Amphid width	13	12 (10–13)	12 (12–13)
Amphid width/cbd (%)	54	48 (38–54)	46 (44–47)
Amphid from anterior end	10	10 (9–11)	11 (9–12)
Nerve ring from anterior end	139	158 (146–178)	168 (134–226)
Nerve ring cbd	69	76 (71–84)	76 (74–78)
Excretory pore from anterior end	196	219 (199–230)	232 (199–268)
Pharynx length	341	347 (311–367)	390 (355–443)
Pharynx bulb cbd	99	94 (87–104)	99 (94–102)
Pharyngeal bulb diameter	62	61 (57–64)	68 (62–74)
Maximum body diameter	102	105 (84–136)	117 (110–127)
Spicule length	108	107 (103–110)	-
Gubernacular apophyses length	47	44 (42–45)	-
Number of precloacal supplements	21	20 or 21	-
Anal or cloacal body diameter	70	88 (84–94)	84 (83–86)
Tail length	190	174 (169–182)	191 (184–199)
V	-	-	1609 (1441–1870)
V%	-	-	51 (48–52)
Vulva body diameter	-	-	119 (117–120)

##### Description.

**Male.** Body long (2628–3093 μm long), quite stout (maximum body width 84–136 μm), narrowing gradually towards both extremities. Cuticle with lateral differentiation of coarser, irregularly spaced punctations starting from amphid to near tail tip. Cuticle striations appearing distinct on cuticle surface under SEM (Fig. 9). Short somatic setae sparsely present on entire body, ~3 μm long. Cephalic region with distinct constriction at level of cephalic setae. Anterior sensilla in three crowns: six small inner labial papillae, six outer labial setae (3 μm long), and four cephalic setae (4–6 μm long or 0.20–0.26 corresponding body diameter long). Anterior buccal cavity cup-shaped, 4–7 μm wide, posterior portion narrow. Spiral amphidial fovea with 2.5 turns, 10–13 μm in diameter or 38–54% of corresponding body diameter, anterior border located at the level of cephalic setae. Pharynx surrounds half of anterior buccal cavity, gradually broadening posteriorly and forming a weak posterior bulb. Nerve ring situated at 40–49% of anterior pharynx. Secretory-excretory gland located posterior to pharynx. Secretory-excretory pore located posterior to nerve ring, 196–230 μm from the anterior end. Cardia small, partially surrounded by intestinal tissue. Reproductive system diorchic, anterior outstretched testis to the left of intestine and posterior outstretched testis to the left or right of the intestine. Spicules arcuate, equal, 1.2–1.5 cloacal body diameters long, proximal part of spicule with central cuticularized projection (lamella) extending from proximal end to ~1/3 of spicule. Gubernaculum with a long straight dorso-caudal apophysis, 42–47 μm long. One precloacal seta 3 μm long, distinct using SEM (Fig. 9C). Twenty or twenty-one precloacal supplements, in the form of small pores, the distance between seven posterior-most supplements more or less equal (10–12 μm), anterior nine supplements with distance between adjacent supplements (12–36 μm) increasing towards anterior (Figs 7, 9). Conico-cylindrical tail 1.8–2.7 cloacal body diameters long with swollen tip bearing three terminal setae. Cylindrical part ~1/5 of total tail length. Three caudal glands present. Spinneret present.

**Female.** Similar to males. Reproductive system with two opposed, outstretched ovaries, with anterior ovary to the left of intestine and posterior ovary to the left or right of intestine. Spermatheca present. Vulva at 48–52% of body length from anterior. Granular vaginal glands present. Three caudal glands present.

##### Diagnosis and relationships.

*Sabatieriacrassilonga* sp. nov. is characterized by relatively stout body (a = 23–33); long body length 2628–3093 μm in males and 2910–3613 μm in females; short cephalic setae (4–6 μm long, 0.20–0.26 corresponding body diameter long); cuticle with lateral differentiation of coarser and irregularly spaced punctations extending from anterior edge of amphid to the tail region; amphidial fovea spiral with 2.5 turns; males with spicules 1.2–1.5 cloacal body diameters long. Twenty or twenty-one fine precloacal supplements. Gubernaculum with a long straight dorso-caudal apophysis, 42–47 μm long. Conico-cylindrical tail 1.8–2.7 cloacal body diameters long in male and 2.1–2.4 cloacal body diameters long in female.

*Sabatieriacrassilonga* sp. nov. belongs to the *praedatrix* group based on amphids with three turns, the presence of pore-like supplements and straight apophysis. An amphid with 2¼, 2½, 2¾, or 3 turns are all considered as three turns (Platt 1985). Within the *praedatrix* group, *Sabatieriacrassilonga* sp. nov. most resembles *S.alata* Warwick, 1973, *S.coomansi* Chen & Vincx, 1999, *S.major* Yang, Guo, Chen & Lin, 2019, *S.triplex* Wieser, 1954, and *S.palmaris* Fadeeva & Belogurov, 1984 in having long body length (2600–4012 μm). The new species can be differentiated from *S.alata* by the ratio of a (23–31 vs 36–53 in *S.alata*) and shorter tail (2.1–2.4 vs 5.5 cloacal body diameters in *S.alata*), from *S.coomansi* by the number of precloacal supplements (20 or 21 vs 23–26 in *S.coomansi*) and shorter tail (2.1–2.4 vs 3.3–3.6 cloacal body diameters in *S.coomansi*), from *S.major* by the shorter body length (2888–3613 μm vs 3879–4255 μm in *S.major*) and the lower value of c’ (1.8–2.7 vs 2.9–3.7 in *S.major*), from *S.triplex* by the ratio of a (23–31 vs 57 in *S.triplex*), length of the cephalic setae (0.20–0.26 vs 0.50 cbd in *S.triplex*) and shorter tail (2.1–2.4 vs 3.7 cloacal body diameters in *S.triplex*), and from *S.palmaris* by the ratio of a (23–31 vs 20–22 in *S.palmaris*) and the ratio of c (16–18 vs 9–11 in *S.palmaris*).

*Sabatieriacrassilonga* sp. nov. is also similar to *S.kelletti* Platt, 1983 in the *celtica* group but differs from the latter by the shorter cephalic setae (0.20–0.26 vs 0.42–0.47 cbd in *S.kelletti*), lower number of precloacal supplements (20 or 21 vs 21–27 in *S.kelletti*) and the lower value of c’ (1.8–2.7 vs 3.6–4.3 in *S.kelletti*).

##### Etymology.

The species is named due to its stout and long body, which derived from Latin *crassa* (= thick, fat, stout) and *longa* (= long).

#### ﻿ Hopperia beaglense

Taxon classificationAnimaliaAraeolaimidaComesomatidae

Chen & Vincx, 1998

E6619A56-CB32-56FB-AF11-B153E5D0178B

**Figure 10.**
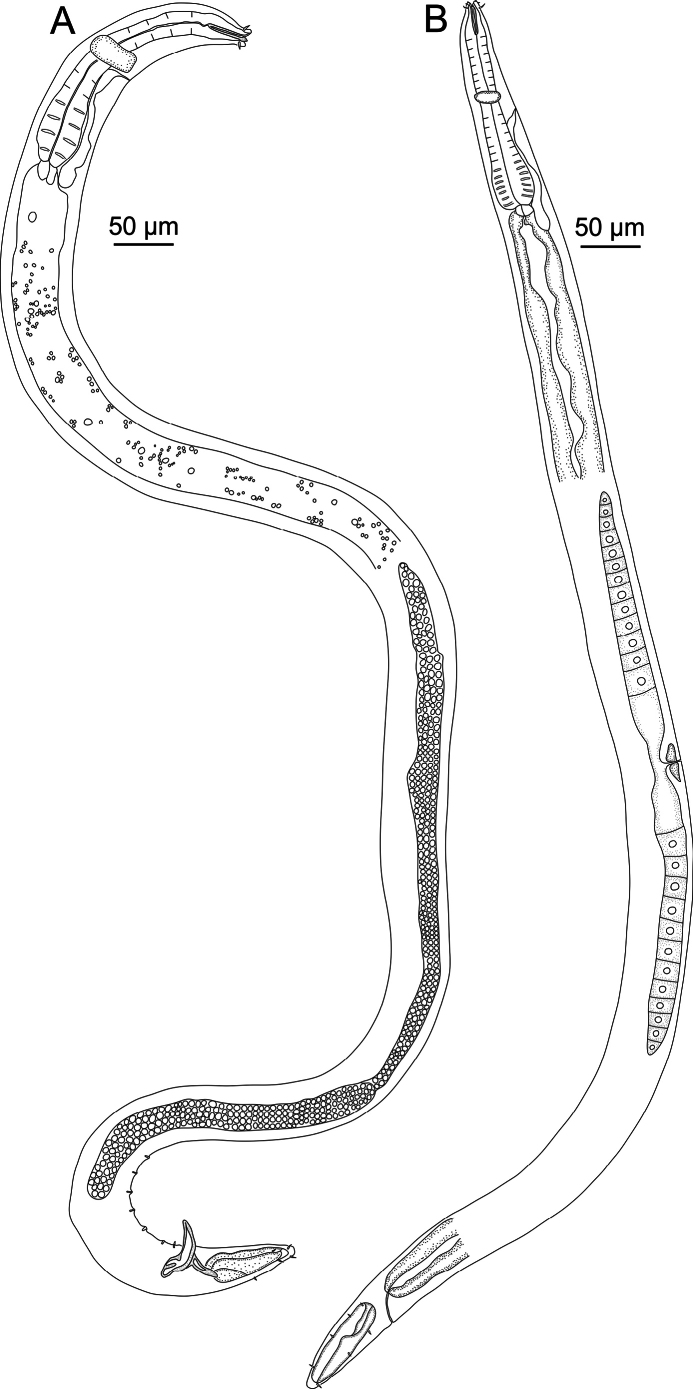
*Hopperiabeaglense* Chen & Vincx, 1998. **A.** Entire male; **B.** Entire female.

**Figure 11.**
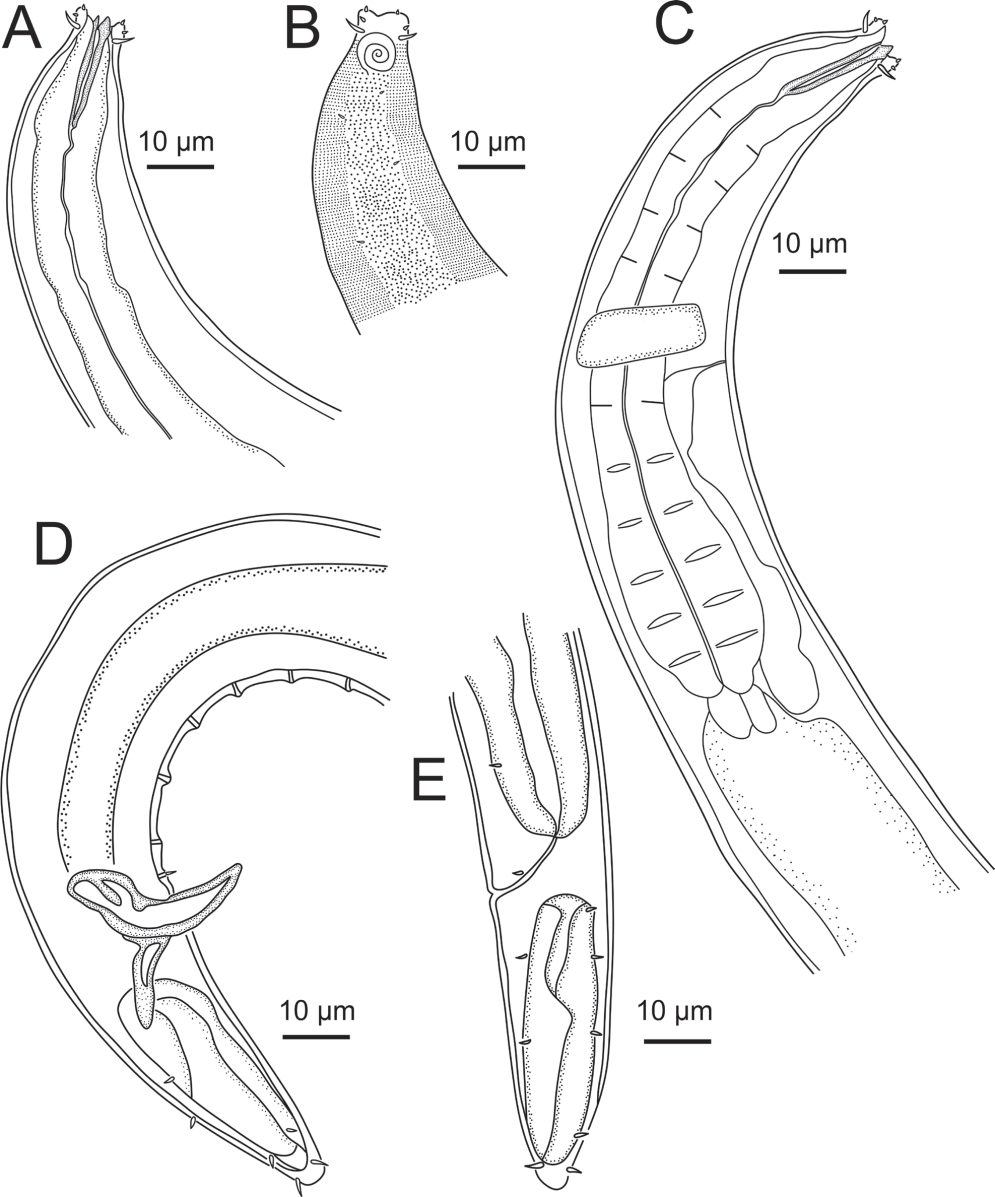
*Hopperiabeaglense* Chen & Vincx, 1998. **A.** Male anterior body region; **B.** Surface view of male anterior body region showing amphidial fovea and cephalic sensilla; **C.** Male pharyngeal body region; **D.** Male posterior body region; **E.** Female posterior body region.

**Figure 12.**
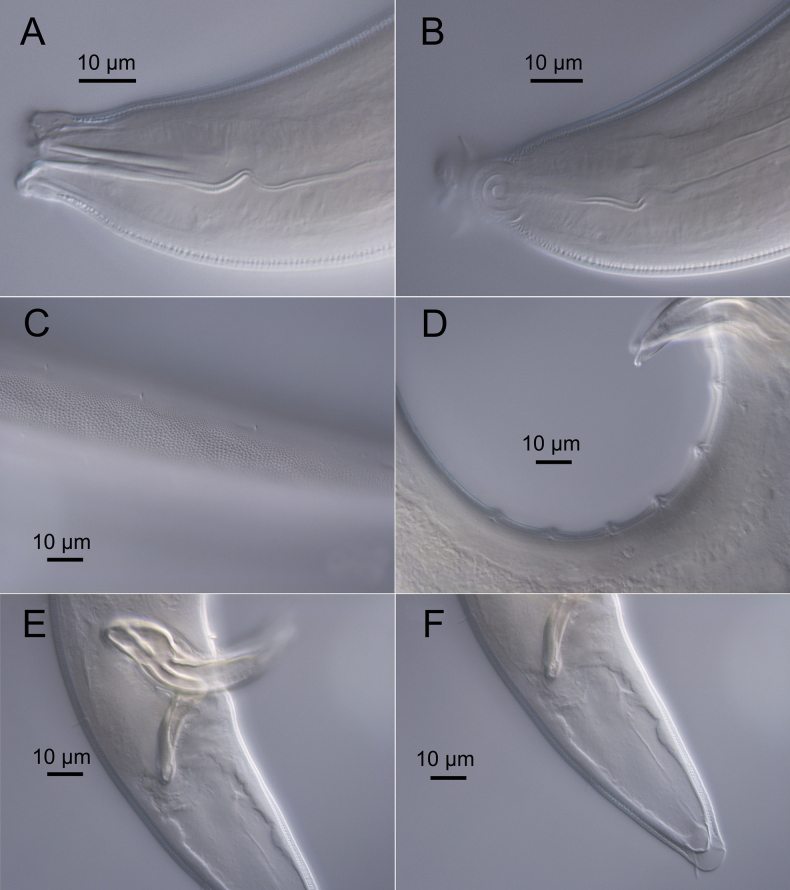
*Hopperiabeaglense* Chen & Vincx, 1998. Light micrographs. **A.** Male anterior part showing buccal cavity; **B.** Surface view of male anterior body region showing amphids; **C.** Lateral punctations of female anterior body region; **D.** Male posterior part showing precloacal supplements; **E.** Male spicular apparatus; **F.** Female tail.

Figs 10 , 11 , 12 , Table 4

##### Material examined.

• Three males, collected in the Prydz Bay, 68.2833°S, 75.5827°E; depth 578 m; collected on 31 Dec. 2020; Jianfeng Mou leg.; total organic carbon 1.37%; sand, 7.87%; clay, 16.77%; silt 75.37%; mean particle diameter 6.20 μm; on slide no. MBSMCOIA-M10D-3-5-10, MBSMCOIA-M10D-1-2-5-4 and MBSMCOIA-M10D-1-5-10-1; female specimen was collected from Antarctic Peninsula, 61.0046°S, 54.6971°W; depth of 574 m; collected on 28 Dec. 2022; Jianfeng Mou leg.; muddy sediment; on slide no. MBSMCOIA-DA-04-2-5-1.

##### Measurements.

All measurement data are given in Table 4.

**Table 4.** Morphometrics (μm) of *Hopperiabeaglense* Chen & Vincx, 1998 from the Southern Ocean.

	Males	Female
Number of specimens	3	1
L	1618–1760	1341
a	20–24	25
b	7-8	6
c	18–21	15
c′	2	3
Head diameter at cephalic setae	14–16	13
Length of cephalic setae	4–5	5
Amphid height	11–12	9
Amphid width	11–12	8
Amphid width/cbd (%)	62–69	54
Amphid from anterior end	7	5
Nerve ring from anterior end	96–109	76
Nerve ring cbd	50–53	36
Excretory pore from anterior end	130–134	131
Pharynx length	226–231	211
Pharynx bulb cbd	56–82	47
Pharyngeal bulb diameter	37–60	33
Maximum body diameter	71–85	54
Spicule length	54–58	-
Gubernacular apophyses length	25–27	-
Anal or cloacal body diameter	42–43	36
Tail length	80–94	89
V	-	678
V%	-	51
Vulva body diameter	-	54

##### Description.

**Male.** Body long (1618–1760 μm long), narrowing gradually towards both extremities. Cuticle with transverse rows of punctations starting from amphid to near tail tip. Lateral differentiation with larger, irregularly spaced punctations. Short somatic setae sparsely present on entire body, ~3 μm long. Cephalic region with distinct constriction at level of cephalic setae. Anterior sensilla in three crowns: six small inner labial papillae, six outer labial setae (2 μm long), and four cephalic setae (4–5 μm long or 0.30–0.33 corresponding body diameter long). Anterior buccal cavity cup-shaped, and the posterior portion cylindrical. Three cuticularized pointed teeth present at the junction between the two compartments of buccal cavity. Spiral amphidial fovea with 3.5 turns, 11–12 μm in diameter or 62–69% of corresponding body diameter, anterior border located at the level of cephalic setae. Pharynx cylindrical, gradually broadening posteriorly and forming a weak posterior bulb. Nerve ring situated at 42–47% of anterior pharynx. Secretory-excretory gland located posterior to pharynx. Secretory-excretory pore located slightly posterior to nerve ring, 96–109 μm from the anterior end. Cardia small, partially surrounded by intestinal tissue. Reproductive system diorchic, anterior outstretched testis to the left or right of intestine and posterior outstretched testis to the right of the intestine. Spicules arcuate, equal, 1.3–1.4 cloacal body diameters long, proximal part of spicule with central cuticularized projection (lamella) extending from proximal end to ~1/4 of spicule. Gubernaculum with slightly curved dorso-caudal apophyses, 25–27 μm long. One precloacal seta 3 μm long. Seven tubular precloacal supplements. Conical tail 1.8–2.2 cloacal body diameters long, two terminal setae (4 μm long). Three caudal glands present.

**Female.** Similar to males, but shorter than males. Reproductive system with two opposed, outstretched ovaries, with anterior ovary to the left of intestine and posterior ovary to the right of intestine. Vulva at 51% of body length from anterior. Granular vaginal glands present. Three caudal glands present.

##### Remarks.

*Hopperiabeaglense* was first described by Chen and Vincx (1998) from the Strait of Magellan and the Beagle Channel, based on specimens collected at 100–110 m water depth with muddy sediment. The present specimens were collected from muddy sediment and at greater water depths (574–578 m). This species differs from all other *Hopperia* species by the conical tail tip. The present specimens are similar to the description by Chen and Vincx (1998), and there are some differences in the value of a (20–25 in present specimens vs 30–41 in the Chilean specimens), maximum body diameter in males (71–85 μm in present specimens vs 41–49 μm in the Chilean specimens), and the present specimens lack the supplement-like structure situated halfway down the ventral side of the tail. This species was also recorded in Kaikoura Canyon, New Zealand (Leduc 2012). So, *H.beaglense* has a circumpolar distribution.

### ﻿Molecular analysis

**Figure 13.**
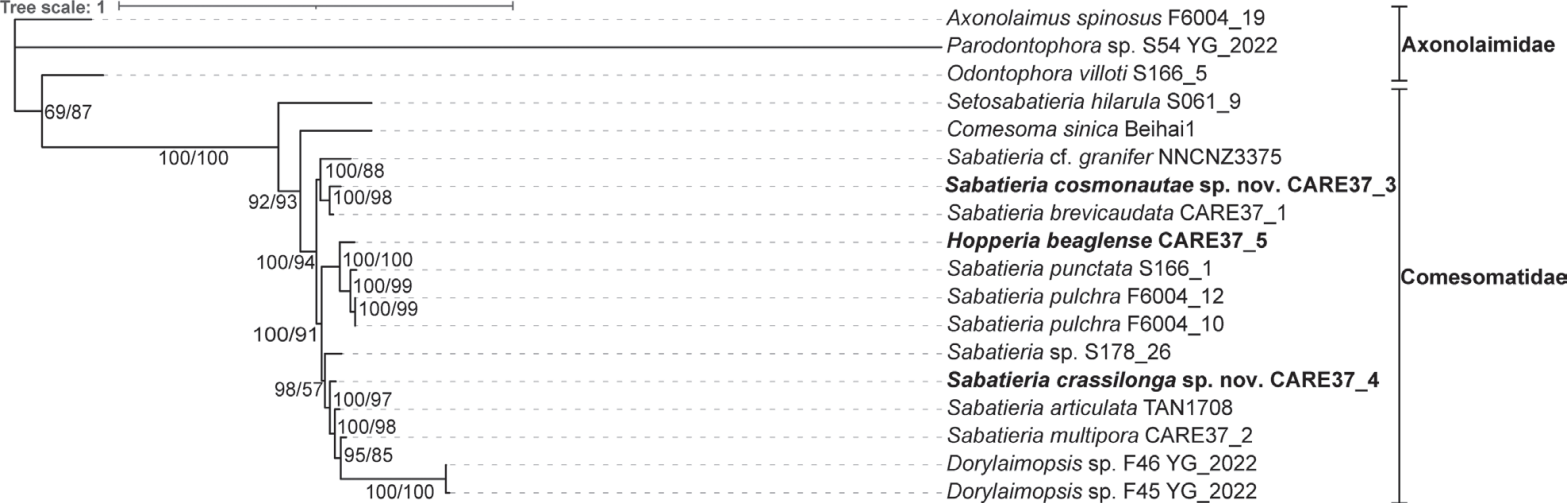
Bayesian inference tree of the families Comesomatidae and Axonolaimidae (outgroup) based on concatenated 18S + 28S rRNA sequences. Posterior probability on the left and bootstrap values on the right are given for the corresponding clades. Sequences obtained in this study are shown in bold. The scale bar represents the number of substitutions per site.

The ML topology trees based on concatenated 18S + 28S rRNA sequences are similar to the BI topology, and only the BI trees are shown (Fig. 13). According to tree topology, 13 species from five genera within the family Comesomatidae, including *Setosabatieria* Platt, 1985, *Comesoma* Bastian, 1865, *Sabatieria*, *Hopperia* Vitiello, 1969 and *Dorylaimopsis* Ditlevsen, 1918, form a well-supported monophyletic clade (100% posterior probability and 100% bootstrap value). Genus *Sabatieria* is not shown as a monophyletic clade which is in accordance with Fu et al. (2019) and Fu et al. (2023) based on 18S rRNA and 28S rRNA sequences. *Sabatieriacosmonautae* sp. nov. was sister to *Sabatieriabrevicaudata* Fu, Zhang, Leduc, Mou & Lin, 2023 (100% posterior probability and 98% bootstrap value). *Sabatieriacrassilonga* sp. nov. was sister to the group of *Sabatieriaarticulata* Fu, Leduc & Zhao, 2019, *Sabatieriamultipora* Fu, Zhang, Leduc, Mou & Lin, 2023 and *Dorylaimopsis* sp. with high support (100% posterior probability and 97% bootstrap value). *Hopperiabeaglense* was sister to the clade consisting of *Sabatieriapunctata* (Kreis, 1924) and *Sabatieriapulchra* (Schneider, 1906) Riemann, 1970 with high support (100% posterior probability and 100% bootstrap value). Table 5 provides the pairwise p-distances for 18S and 28S rRNA sequences. Within the Comesomatidae, genetic distances between *Sabatieriacosmonautae* sp. nov. and other species ranged from 0.87% to 10.79% for 18S rRNA sequences, and from 0.15% to 25.82% for 28S rRNA; genetic distances between *Sabatieriacrassilonga* sp. nov. and other species ranged from 0.29% to 10.79% for 18S rRNA sequences, and from 0 to 26.78% for 28S rRNA sequences. Relationship analysis based on 18S rRNA sometimes contradicts the results from 28S rRNA (Liang et al. 2024), which makes determining the relationships among nematodes even more complex. Phylogenetic analysis based on concatenated 18S + 28S rRNA sequences might give more hints to solve relationships among nematodes. Besides accurate morphological identification, phylogenetic relationships based on concatenated rRNA sequence analysis requires obtaining multiple gene fragments from the same specimen. Due to the limited availability of gene data from the same specimen in GenBank, we could not confirm whether concatenated 18S + 28S rRNA sequence analysis is superior to the analysis of a single gene fragment. DNA barcoding is aiding in species identification nowadays, and more nuclear and mitochondrial genes are needed to obtain more reliable results in phylogenetic relationships of nematodes.

**Table 5.** Pairwise p-distance among the 18S and 28S rRNA sequences of Comesomatidae (the matrix in the lower left and upper right among the 18S rRNA sequences and 28S rRNA sequences).

		1	2	3	4	5	6	7	8	9	10	11	12	13	14	15
1	*Sabatieriacosmonautae* sp. nov. CARE37_3		0.0044	0.1464	0.0044	0.0015	0.1252	0.1118	0.1426	0.1426	0.1349	0.1040	0.2177	0.2505	0.2582	0.2582
2	*Sabatieriacrassilonga* sp. nov. CARE37_4	0.1079		0.1252	0.0000	0.0983	0.0559	0.0809	0.1310	0.1310	0.1310	0.1214	0.2235	0.2678	0.2177	0.2177
3	*Hopperiabeaglense* CARE37_5	0.0116	0.0073		0.0073	0.0102	0.0087	0.0116	0.0058	0.0058	0.0087	0.1561	0.2216	0.2563	0.2158	0.2158
4	*Sabatieriaarticulata* TAN1708	0.1021	0.0520	0.1233		0.0906	0.0405	0.0771	0.1118	0.1118	0.1195	0.1137	0.2274	0.2543	0.2023	0.2023
5	*Sabatieriabrevicaudata* CARE37_1	0.0347	0.0029	0.1291	0.0029		0.1156	0.0867	0.1387	0.1387	0.1426	0.1002	0.2100	0.2524	0.2524	0.2524
6	*Sabatieriamultipora* CARE37_2	0.0087	0.0044	0.1195	0.0044	0.0073		0.0944	0.1214	0.1214	0.1252	0.1329	0.2274	0.2640	0.1965	0.1965
7	*Sabatieria* sp. S178_26	0.0087	0.0073	0.1233	0.0073	0.0073	0.0116		0.1195	0.1195	0.1272	0.1040	0.2351	0.2717	0.2312	0.2312
8	*Sabatieriapulchra* F6004_10	0.0116	0.0073	0.0925	0.0073	0.0102	0.0087	0.0116		0.0000	0.0385	0.1541	0.2370	0.2601	0.2447	0.2447
9	*Sabatieriapulchra* F6004_12	0.0116	0.0073	0.0925	0.0073	0.0102	0.0087	0.0116	0.0000		0.0385	0.1541	0.2370	0.2601	0.2447	0.2447
10	*Sabatieriapunctata* S166_1	0.0116	0.0102	0.0809	0.0102	0.0102	0.0116	0.0087	0.0058	0.0058		0.1618	0.2351	0.2640	0.2274	0.2274
11	Sabatieriacf.granifer NNCNZ3375	0.0102	0.0087	0.0160	0.0087	0.0087	0.0116	0.0131	0.0131	0.0131	0.0131		0.2216	0.2582	0.2331	0.2331
12	*Comesomasinica* Beihai1	0.0247	0.0203	0.0247	0.0203	0.0233	0.0233	0.0218	0.0218	0.0218	0.0247	0.0203		0.2832	0.2678	0.2678
13	*Setosabatieriahilarula* S061_9	0.0305	0.0320	0.0349	0.0320	0.0291	0.0363	0.0334	0.0363	0.0363	0.0363	0.0378	0.0465		0.3295	0.3295
14	*Dorylaimopsis* sp. F45_YG_2022	0.0378	0.0363	0.0407	0.0363	0.0363	0.0349	0.0378	0.0363	0.0363	0.0378	0.0422	0.0480	0.0596		0.0000
15	*Dorylaimopsis* sp. F46_YG_2022	0.0276	0.0262	0.0305	0.0262	0.0262	0.0247	0.0276	0.0262	0.0262	0.0276	0.0320	0.0378	0.0494	0.0116	

## Supplementary Material

XML Treatment for Sabatieria

XML Treatment for Sabatieria cosmonautae

XML Treatment for Sabatieria crassilonga

XML Treatment for Hopperia beaglense

## ﻿Additional information

### Use of AI

No use of AI was reported.

### Author ORCIDs

Sujing Fu https://orcid.org/0000-0002-0061-0955

Jianfeng Mou https://orcid.org/0000-0002-4432-5814

Kun Liu https://orcid.org/0009-0008-3923-7159

Shuyi Zhang https://orcid.org/0009-0008-7114-9159

Heshan Lin https://orcid.org/0000-0001-9286-9421

### Data availability

All of the data that support the findings of this study are available in the main text.
